# Exploring the hepatoprotective properties of citronellol: *In vitro* and *in silico* studies on ethanol-induced damage in HepG2 cells

**DOI:** 10.1515/biol-2022-0950

**Published:** 2024-09-09

**Authors:** Muhammad Nasir Hayat Malik, Iqra Abid, Sana Ismail, Irfan Anjum, Halima Qadir, Tahir Maqbool, Komal Najam, Samir Ibenmoussa, Mohammed Bourhia, Ahmad Mohammad Salamatullah, Gezahign Fentahun Wondmie

**Affiliations:** Faculty of Pharmacy, The University of Lahore, Lahore, Pakistan; Department of Basic Medical Sciences, Shifa College of Pharmaceutical Sciences, Shifa Tameer-e-Millat University, Islamabad, Pakistan; Institute of Molecular Biology and Biotechnology, The University of Lahore, Lahore, Pakistan; Laboratory of Therapeutic and Organic Chemistry, Faculty of Pharmacy, University of Montpellier, Montpellier, 34000, France; Laboratory of Biotechnology and Natural Resources Valorization, Faculty of Sciences, Ibn Zohr University, 80060, Agadir, Morocco; Department of Food Science & Nutrition, College of Food and Agricultural Sciences, King Saud University, 11 P.O. Box 2460, Riyadh, 11451, Saudi Arabia; Department of Biology, Bahir Dar University, P.O.Box 79, Bahir Dar, Ethiopia

**Keywords:** citronellol, hepatoprotective, cell viability, HepG2 cell lines

## Abstract

Citronellol (CT) is a monoterpene alcohol present in the essential oil of plants of the genus *Cymbopogon* and exhibits diverse pharmacological activities. The aim of the current study was to investigate the hepatoprotective potential of CT against ethanol-induced toxicity in HepG2 cell lines. Silymarin (SIL) was used as a standard drug. MTT, crystal violet assay, DAPI, and PI staining were carried out to assess the effect of ethanol and CT on cell viability. RT-PCR determined the molecular mechanisms of hepatoprotective action of CT. CT ameliorated cell viability and restricted ethanol-induced cell death. DAPI and PI staining showed distinct differences in cell number and morphology. Less cell viability was observed in the diseased group obviously from strong PI staining when compared to the CT- and SIL-treated group. Moreover, CT showed downregulation of interleukin (IL-6), transforming growth factor-beta 1 (TGF-β1), collagen type 1 A 1 (COL1A1), matrix metalloproteinase-1 (MMP-1), tissue inhibitor of metalloproteinase-1 (TIMP-1) and glutathione peroxidase-7 (GPX-7) levels. Molecular docking studies supported the biochemical findings. It is concluded that the cytoprotective activity of CT against ethanol-induced toxicity might be explained by its anti-inflammatory, immunomodulatory, and collagen-regulating effects.

## 1 Introduction

The liver is the principal organ of the body implicated in regulating glucose, lipids, amino acids, and energy metabolism. Besides, it produces blood proteins that carry steroids, fatty acids, vitamins, amino acids, and pharmacological agents and are responsible for regulating blood clotting factors and osmotic pressure and immunoglobin that provide natural defense to the body [1,2,3,4]. Many hazardous compounds are detoxified and transformed by the liver for removal through the kidney in the form of urine or as bile via the colon. Liver enzymes convert various drugs to active or inactive state [5]. Therefore, any damage to the liver can have severe consequences, as no other organ in the body can compensate for its functional loss [6].

Liver damage has been considered one of the key priority concerns in healthcare. Approximately 500 million individuals worldwide are afflicted with a severe manifestation of liver problems, as estimated by the World Health Organization [7]. Hepatic diseases cause the death of thousands of individuals worldwide each year. Approximately 2 million individuals succumb to liver disease annually, with 1 million deaths attributed to complications of cirrhosis and another 1 million resulting from viral hepatitis and hepatocellular carcinoma [5]. A wide range of metabolic, chemical, and inflammatory stressors can induce necrosis, apoptosis, and autophagy activation in liver cells leading to its damage. The occurrence of cell death in distinct cell types can contribute to the development of different liver disorders [8,9].

The hepatoprotective potential of medicinal plants and their phytocompounds has been reported in various studies [10,11,12,13]. Herbal medicine is preferred over conventional drugs for several reasons: it is less expensive; it causes less adverse reactions and is comparatively safer [7,14]. Citronellol (CT) is a monoterpene alcohol found in the essential oil of plants of the genus Cymbopogon and possesses a variety of medicinal properties [15,16,17] including antibacterial, antifungal, antispasmodic, and anticonvulsant [18]. Considering the demand for new efficacious drugs and the documented therapeutic effects of CT, it is plausible to speculate that CT might serve as a promising compound to focus on in further pharmacological studies. In vitro bioassays are important in the evaluation of plants with possible hepatoprotective effects [19]. Therefore, the aim of this work was to discover the hepatoprotective potential of CT in the HepG2 cell line and also to investigate the underlying molecular pathways of CT’s hepatoprotective activity.

## 2 Materials and methods

### 2.1 HepG2 cell line

HepG2 cell lines are widely employed to assess the hepatoprotective properties of compounds [19,20,21]. HepG2 cell lines were provided by the Cell and Tissue Culture Laboratory of The University of Lahore. Cell lines were revived from cryovials that had been stored in liquid nitrogen.

Dulbecco’s modified Eagle’s medium (DMEM) containing phenol red supplemented with 10 mM HEPES buffer, 100 IU/mL penicillin and 100 μg/mL streptomycin (ATCC), and 10% fetal bovine serum (ATCC) at 37°C under a 5% carbon dioxide humidified atmosphere were used to culture hepG2 cell lines. Every 3–4 days, the growth medium was changed. When 80% confluency was attained, after washing with calcium and magnesium-free phosphate-buffered saline (PBS) solution, cells were trypsinized with EDTA and then collected by centrifugation and re-suspended in a fresh growth medium [22].

### 2.2 Cytotoxicity evaluation of ethanol and CT

Cell viability assay was conducted to find the optimal concentrations of CT and ethanol. Different concentrations of ethanol (1, 3, 5, 8, and 10%) were prepared in complete DMEM and 1 M stock solution of CT was prepared by dissolving in DMSO. Several dilutions of CT (5, 10, 50, 100, 500, and 1,000 µM) were later formulated from 1 M stock solution, with a final DMSO concentration of less than 0.1%. HepG2 cells were seeded on a 96-well plate and incubated at 37°C overnight. The next day, the medium from the wells was withdrawn and the cells were washed with 1× PBS. 100 µL of different concentrations of ethanol and CT were introduced into the wells for 24 h. Cell viability of treated cells were assessed by MTT assay according to protocol [20].

### 2.3 Cytoprotective effect of CT against EtOH-induced damage

Cells were pretreated with different concentrations of CT (10–500 µM) for 24 h and subsequently treated with 8% ethanol. Cell viability of treated cells was assessed by MTT and crystal violet assays according to standard protocols [20].

HepG2 cells were divided into the following groups:

1. Control: No treatment
2. DMSO control: 10% DMSO solution in DMEM
3. Disease control: 8% EtOH in DMEM
4. Silymarin (SIL) 200 µg/mL: SIL (200 µg/mL) followed by 8% EtOH in DMEM
5. CT 10 µM: CT (10 µM) followed by 8% EtOH in DMEM
6. CT 50 µM: CT (50 µM) followed by 8% EtOH in DMEM
7. CT 100 µM: CT (100 µM) followed by 8% EtOH in DMEM
8. CT 500 µM: CT (500 µM) followed by 8% EtOH in DMEM

### 2.4 MTT assay

Pretreated cells were washed with 1× PBS followed by 3–4 h of incubation with 100 µL of DMEM and 25 µL of MTT solution. Formazan crystals were solubilized with 10% sodium dodecyl sulfate (SDS), and absorbance at 570 nm was measured using a microplate reader. Cell viability percentage was calculated from the mean absorbance values. SIL was used as a reference drug [21].

### 2.5 Crystal violet assay

Pretreated cells were rinsed with 1× PBS and treated with a mixture of 0.1% crystal violet dye and 2% ethanol followed by incubation for 15 min at room temperature. Wells were thoroughly washed with 1× PBS and the dye was carefully disposed off. The stain was then solubilized by adding 100 µL of 1% SDS to each well and being allowed for 5–10 min at room temperature. Finally, absorbance at 595 nm was measured using a microplate reader.

### 2.6 Real-time PCR analysis

The conventional TRIzol technique was used to extract total RNA from HepG2 cells which was later reverse transcribed according to standard protocol using cDNA kit. Relative mRNA expressions of indicated genes were measured by the ΔΔCT method; HPRT was used as housekeeping control. The forward and reverse primers used are listed in Table 1.

**Table 1:** Primers with their sequences

Gene	Primer	Sequence
IL-6	IL-6_F	5′-ACCTGAACCTTCCAAAGATG-3′
	IL-6_R	5′-GCTTGTTCCTCACTACTCTC-3′
COL1A1	COL1A1_F	5′-AAGCAACCCAAACTGAACCC-3′
	COL1A1_R	5′-TTCAAGCAAGTGGACCAAGC-3′
TGF-β1	TGF-β1_F	5′-TTGAGACTTTTCCGTTGCCG-3′
	TGF-β1_R	5′-CGAGGTCTGGGGAAAAGTCT-3′
MMP-1	MMP1_F	5′-CTGGCCACAACTGCCAAATG-3′
	MMP1_R	5′-CTGTCCCTGAACAGCCCAGTACTTA-3′
TIMP-1	TIMP1_F	5′-CCTTCTGCAATTCCGACCTC-3′
	TIMP1_R	5′-GTATCCGCAGACACTCTCCA-3′
GPX-7	GPX7_F	5′-AACTGGTGTCGCTGGAGAAG-3′
	GPX7_R	5′-AAACTGGTTGCAGGGGAAG-3′
HPRT	HPRT_F	5′-GAACGTCTTGCTCGAGATGTG-3′
	HPRT_R	5′-CCAGCAGGTCAGCAAAGAATT-3′

### 2.7 *In silico* studies

#### 2.7.1 Protein structure

**Figure 1:**
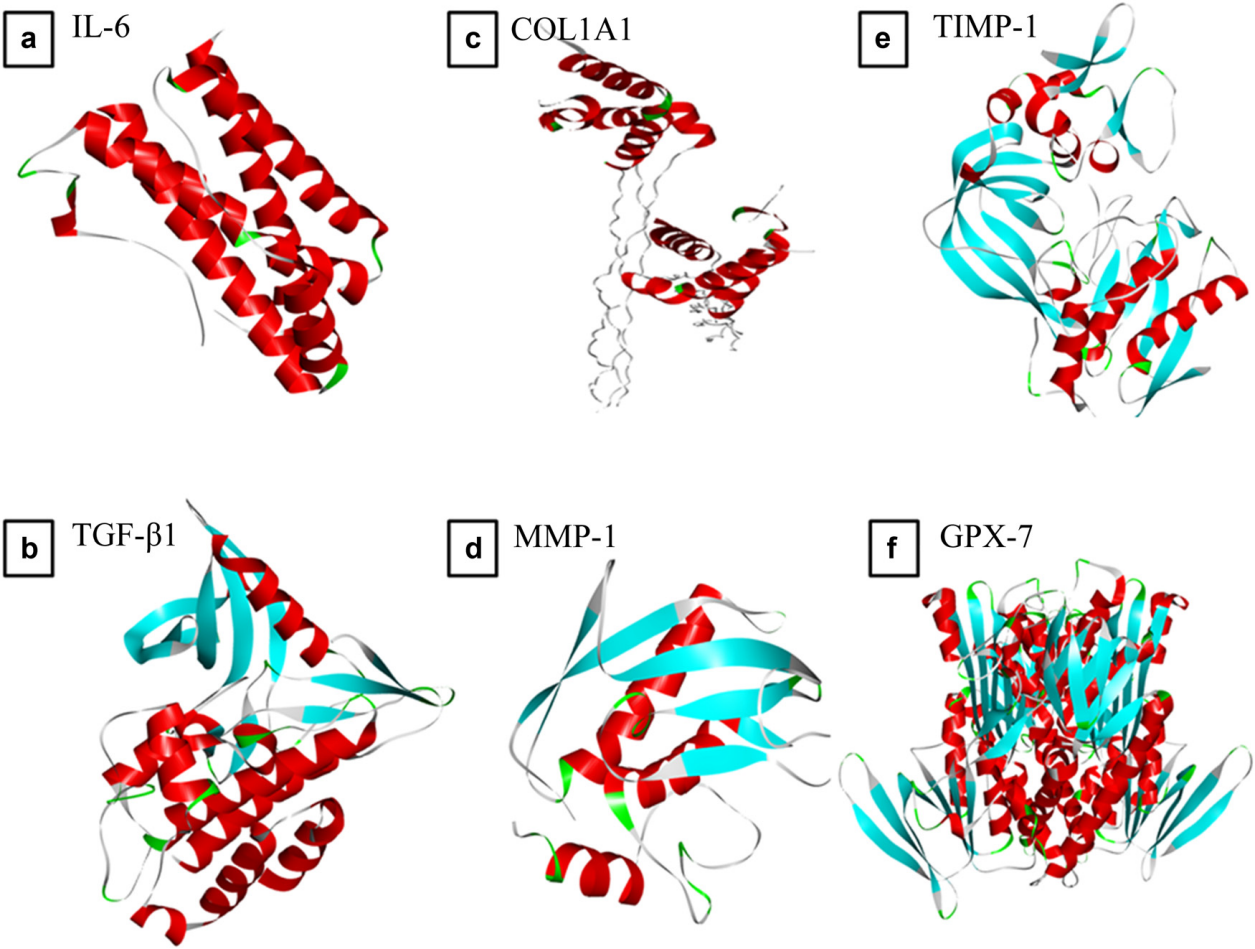
X-ray crystallographic structure of (a) IL-6 (1ALU), (b) TGF-β1 (3KFD), (c) COL1A1 (5cvb), (d) MMP-1 (1hfc), (e) TIMP-1 (2e2d), and (f) GPX-7 (2gh5).

The X-ray crystallography structures of interleukin (IL-6) (1ALU), transforming growth factor-beta 1 (TGF-β1) (3KFD), COL1A1 (5cvb), MMP-1 (1hfc), TIMP-1 (2e2d), and GPX-7 (2gh5) derived from homo sapiens were downloaded from RCSB PDB (https://www.rcsb.org/) (Figure 1) [23]. The active sites of each target protein were identified through the BIOVIA Discovery Studio Visualizer. Target proteins were prepared by removing ligands and water molecules and adding polar hydrogen bonds. The altered protein file was saved in .pdb format. The modified protein structure files and downloaded chemical structures were used as inputs in PyRx 0.8 program.

#### 2.7.2 Ligand structures

The three-dimensional (3D) structure of ligands CT (CID-8842) and SIL (CID-5213) was retrieved in sdf format from PubChem (https://pubchem.ncbi.nlm.nih.gov/). These downloaded structures undergo energy minimization in the universal force field and are subsequently converted to pdbqt format.

### 2.8 Molecular docking

Vina Wizard in PyRx was used for molecular docking. Target proteins were loaded as macromolecules. Exhaustiveness was maintained at 8 while other parameters were set as default. The grid box was adjusted to cover all active sites in each protein. After protein–ligand docking, the binding affinity (kcal/mol) obtained was exported as comma-separated values files. The visualization diagram for both two and three dimensions was constructed through BIOVIA Discovery Studio to analyze protein–ligand complex interactions [23].

### 2.9 Statistical analysis

Data of 3–4 biological replicates were expressed as mean ± standard deviation and were analyzed by one-way analysis of variance (ANOVA) followed by Tukey’s multiple comparison test. All statistical analyses were carried out by using GraphPad Prism 8.0 software. The probability of less than 0.05 was considered significant. The following abbreviations were used to demonstrate significance in the graphs: *p < 0.05, **p < 0.01, ***p < 0.001.

### 2.10 Results CT showed no impact on cell viability

**Figure 2:**
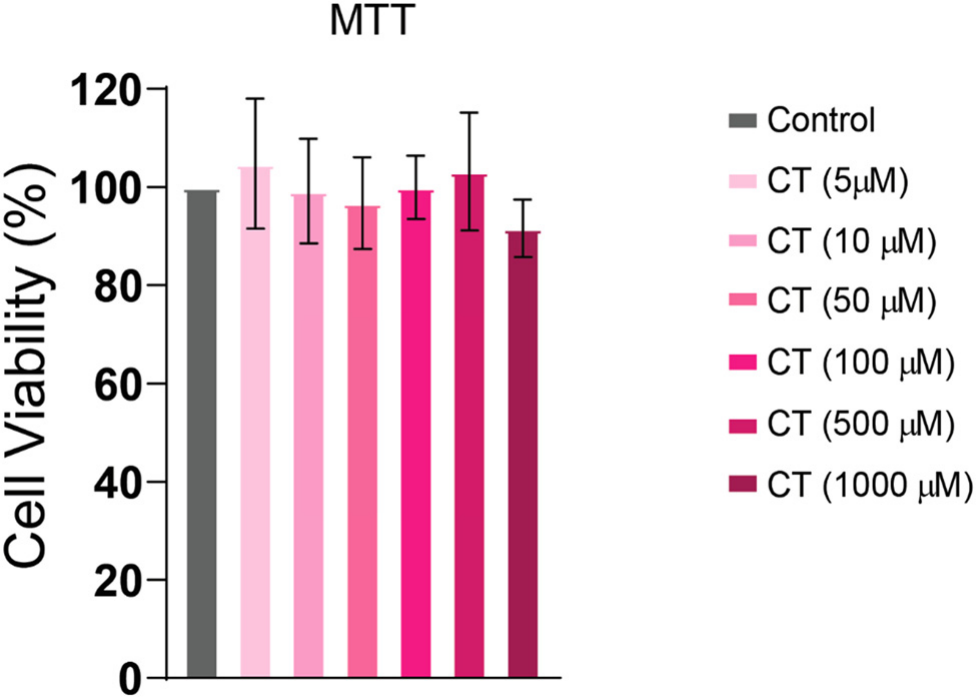
Cell viability at different doses of CT. HepG2 cells were treated with increasing doses of CT (10–1,000 µM) for 24 h. CT did not display any significant reduction in cell viability at the observed concentrations. Control group: no treatment.

MTT assay revealed no impact of CT on cell viability at the indicated doses. At a dose of 1,000 µM, a slight reduction in cell viability was observed indicating that doses above 1,000 µM could be toxic to HepG2 cells (Figure 2).

### 2.11 Cytotoxic effect of ethanol on HepG2 cells

**Figure 3:**
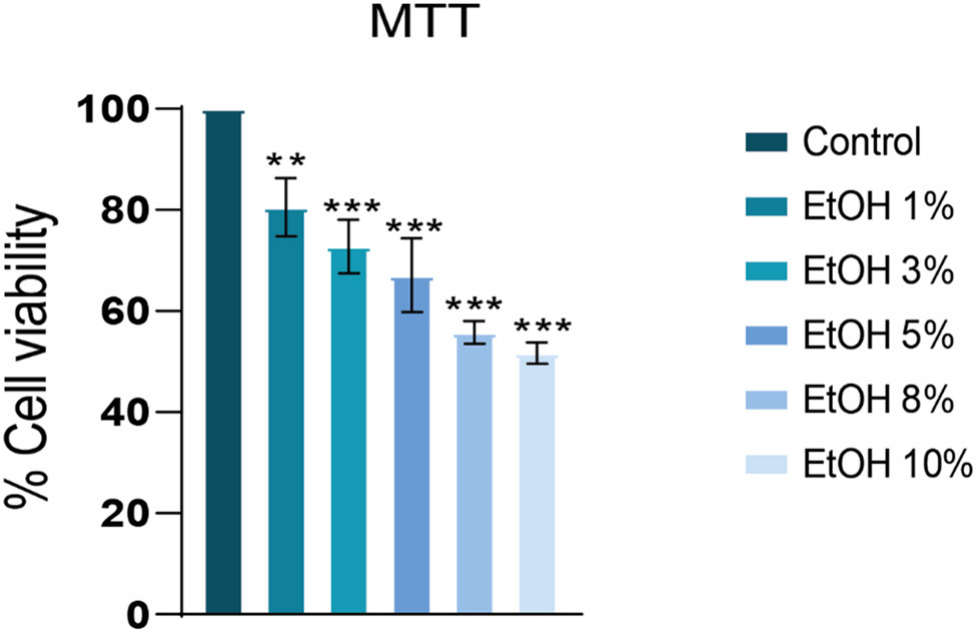
Cytotoxicity of ethanol at various doses in HepG2 cell line. Cells were exposed to increasing ethanol concentrations for 24 h. EtOH displayed a dose-dependent reduction in cell viability. **p < 0.01, ***p < 0.001 (one-way ANOVA by Tukey’s multiple comparison test). Control group: no treatment.

The cells were exposed to increasing concentrations of ethanol (1, 3, 5, 8, and 10%) for 24 h to determine ethanol toxicity. Ethanol showed toxicity at almost all concentrations with an IC50 value of 12.37%. 1% showed a slight reduction in cell viability, while 3 and 5% ethanol reduced cell viability by more than 60%. Congruently, 8 and 10% concentrations of ethanol decreased the cell viability by more than 50% (Figure 3).

#### 2.11.1 CT mitigates ethanol-induced toxicity

**Figure 4:**
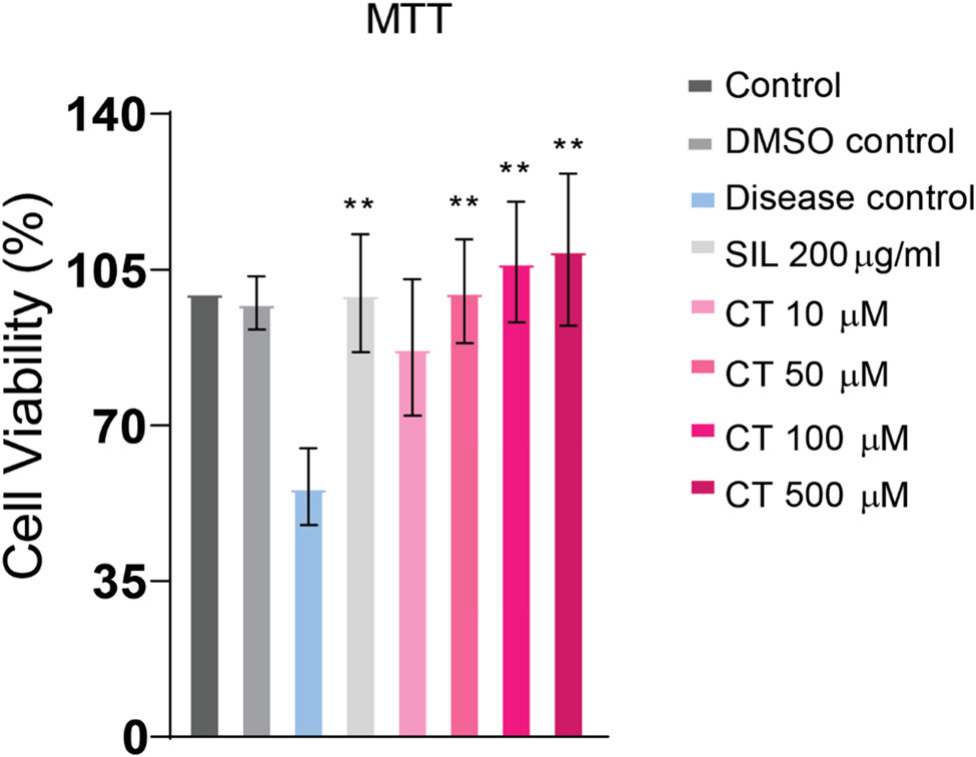
CT reduced ethanol-induced cytotoxicity in HepG2 cell line. HepG2 cells were pretreated with CT for 24 h followed by ethanol (8%) intoxication for 24 h. Pretreatment with CT at doses of 10, 50, 100, and 500 µM dramatically reduced ethanol intoxication and increased cell survival. **p < 0.01 (one-way ANOVA by Tukey’s multiple comparison test). Control group: no treatment, DMSO control: 10% DMSO, SIL: silymarin treatment, CT: citronellol treatment.

CT’s cytoprotective efficacy against ethanol-induced cell damage was examined at doses of 10, 50, 100, and 500 µM. These doses of CT significantly prevented ethanol-mediated reduction in cell viability indicating its cytoprotective effect against ethanol toxicity (Figure 4).

### 2.12 Crystal violet assay demonstrated hepatoprotective effect of CT

**Figure 5:**
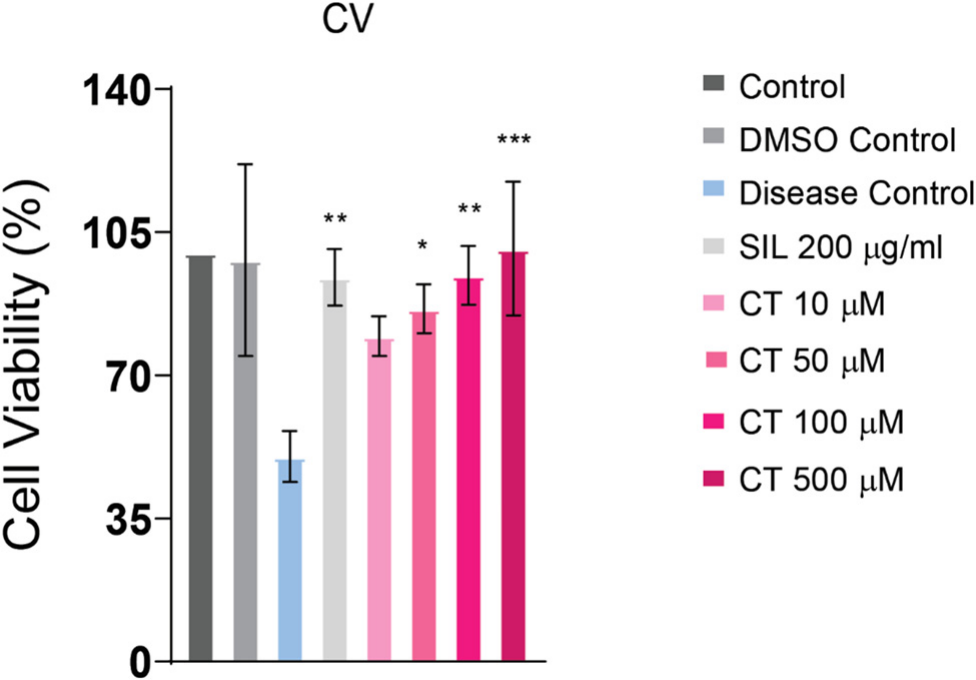
Crystal violet assay displaying the degree of cell survival in various treated and untreated groups. HepG2 cells were pretreated with CT for 24 h followed by ethanol (8%) intoxication for 24 h. In comparison to the disease group, cell viability was enhanced in the CT pre-treated group, which demonstrated that CT protected HepG2 cells from ethanol toxicity. *p < 0.05, **p < 0.01, ***p < 0.001 (one-way ANOVA by Tukey’s multiple comparison test). Control group: no treatment, DMSO control: 10% DMSO, SIL: silymarin treatment, CT: citronellol treatment.

Crystal violet was used to analyze cell viability. In contrast to the disease group, crystal violet analysis of CT- and SIL-treated cells showed reduced cell death. The ethanol-induced cell toxicity in CT-treated cells decreased dose dependently (Figure 5).

### 2.13 DAPI and PI fluorescent staining showed reduced cell death in CT-treated groups

**Figure 6:**
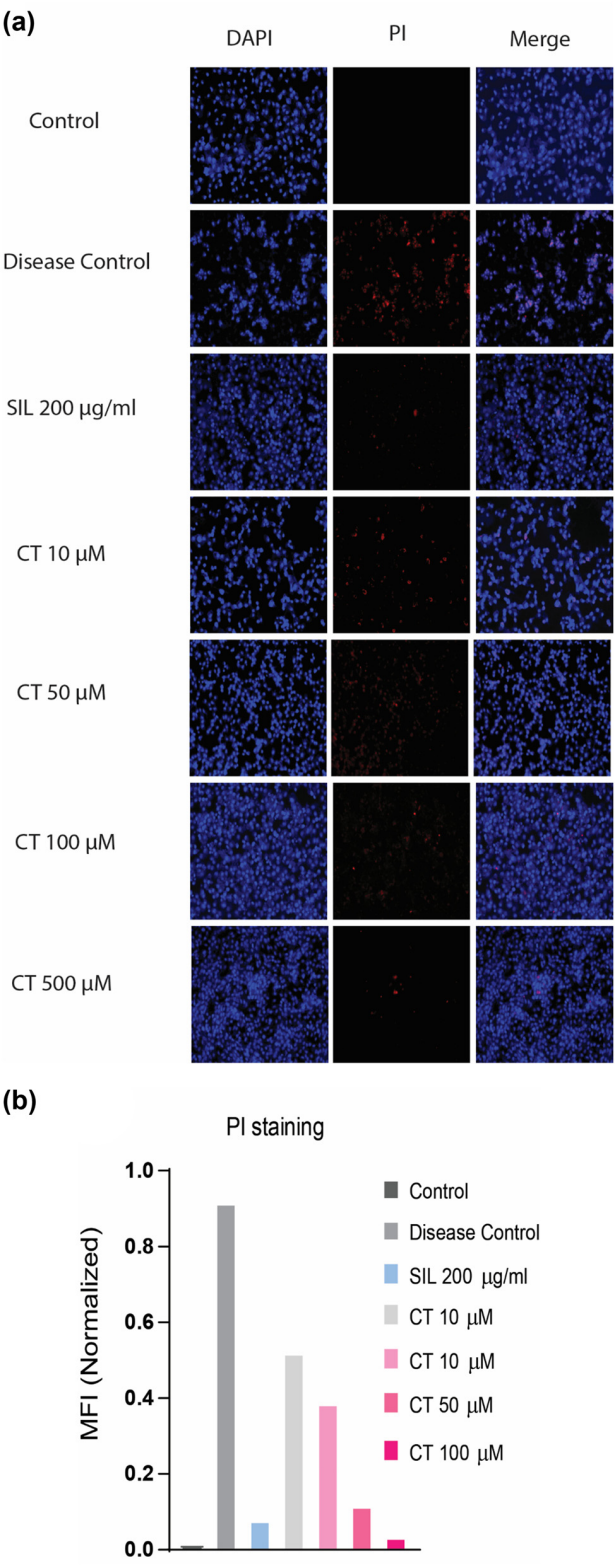
DAPI and PI staining demonstrated a reduction in ethanol-induced cell death in CT-treated cells. Cells were pretreated with indicated concentrations of CT followed by EtOH intoxication. Subsequently, cells were stained with DAPI and PI to measure cell damage and cell death. Stained cells were photographed by inverted microscope (a) and were quantified by Image J software. Mean fluorescence intensities (MFI) of DAPI- and PI-stained cells were determined and results were expressed as normalized MFI values (b). Normalized MFI value = MFI PI/MFI DAPI.

PI stain is used to stain dead cells. The damaged cell membrane of dead cells allows the PI dye to enter the cell where it may attach to the DNA and produces fluorescence, allowing the identification of the dead cells [24]. The staining revealed distinct differences in cell number and morphology. The disease control group’s cells were smaller, fewer in number, and stained more heavily with PI than CT- and SIL-treated groups. CT at a dose of 500 µM displayed a significant number of live cells comparable to SIL treated group (Figure 6).

### 2.14 CT suppressed mRNA levels of inflammatory and collagen-inducing genes

**Figure 7:**
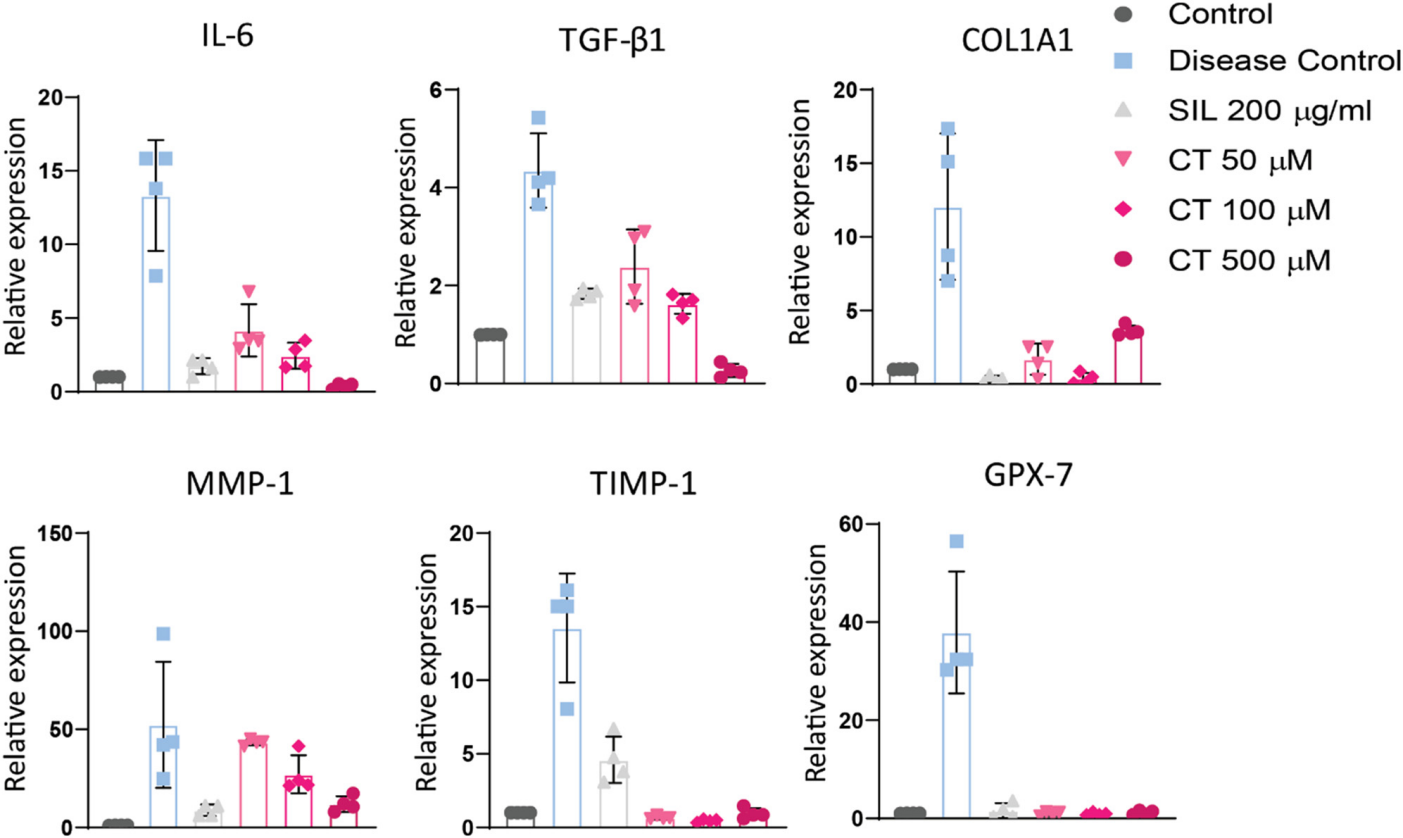
CT demonstrated hepatoprotective effects via suppression of inflammatory and collagen-inducing genes. HepG2 cells were pretreated with CT for 24 h, and later, these were exposed to ethanol (8%) for 24 h to induce injury. RT-PCR was carried out to examine relative expression by the ΔΔCT method. A prominent down-regulation of biomarkers was observed in cells pretreated with CT and SIL. Control group: no treatment, SIL: silymarin treatment, CT: citronellol treatment.

To investigate the molecular mechanism of CT’s hepatoprotective action against ethanol-induced cytotoxicity, the relative mRNA expression of biomarkers such as IL-6, TGF-B1, COL1A1, MIMP-1, TIMP-1, and GPX-7 was assessed. The data showed a remarkable reduction of these biomarkers in CT-treated cells as compared to the disease group. The reduction in biomarkers in CT-treated cells was comparable to the standard drug SIL indicating hepatoprotective propensity of CT (Figure 7).

### 2.15 Analysis of molecular docking

**Figure 8:**
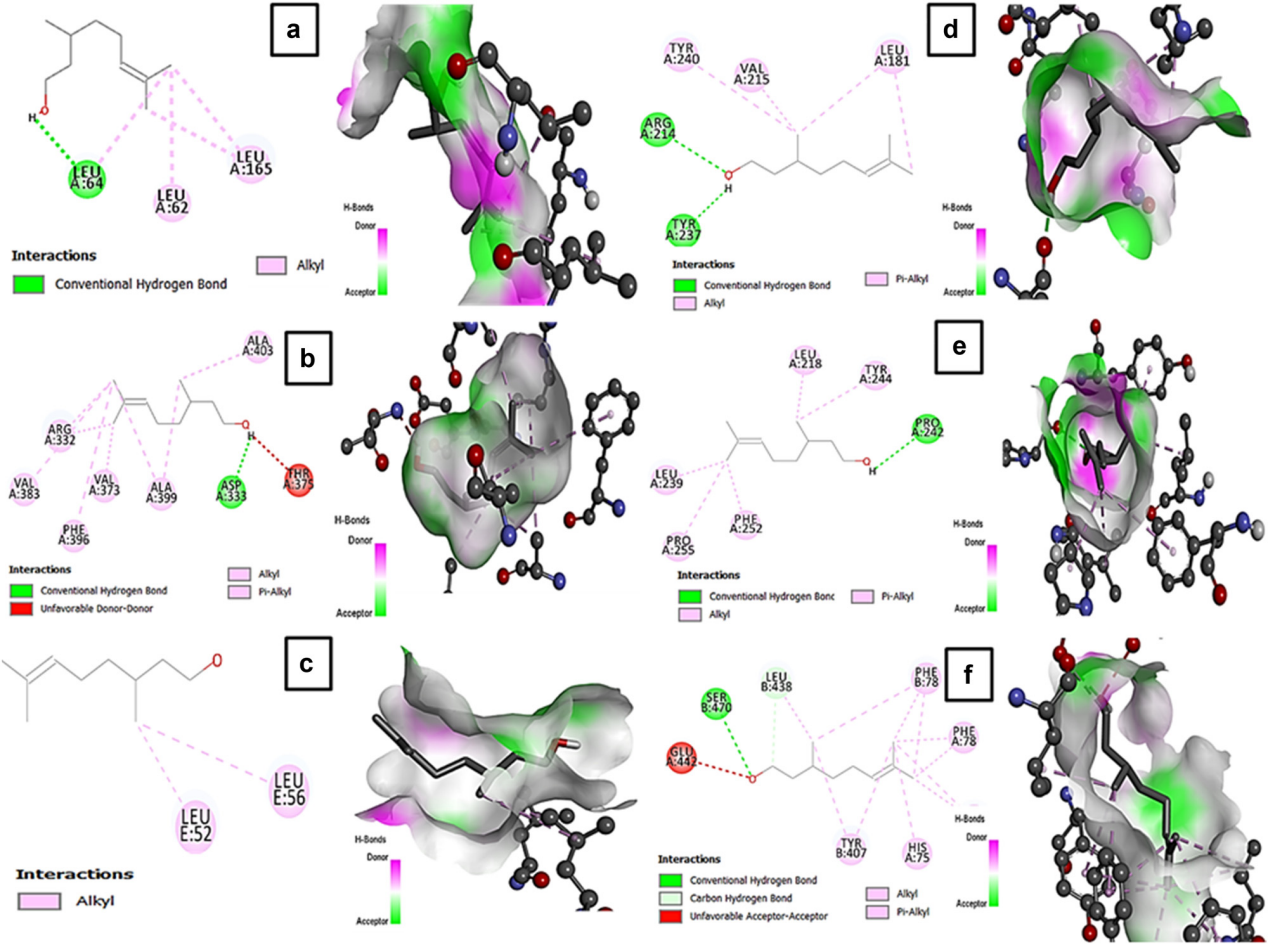
2D and 3D interaction of CT with target proteins (a) IL-6 (1ALU), (b) TGF-β1 (3KFD), (c) COL1A1 (5cvb), (d) MMP-1 (1hfc), (e) TIMP-1 (2e2d), and (f) GPX-7 (2gh5).

**Figure 9:**
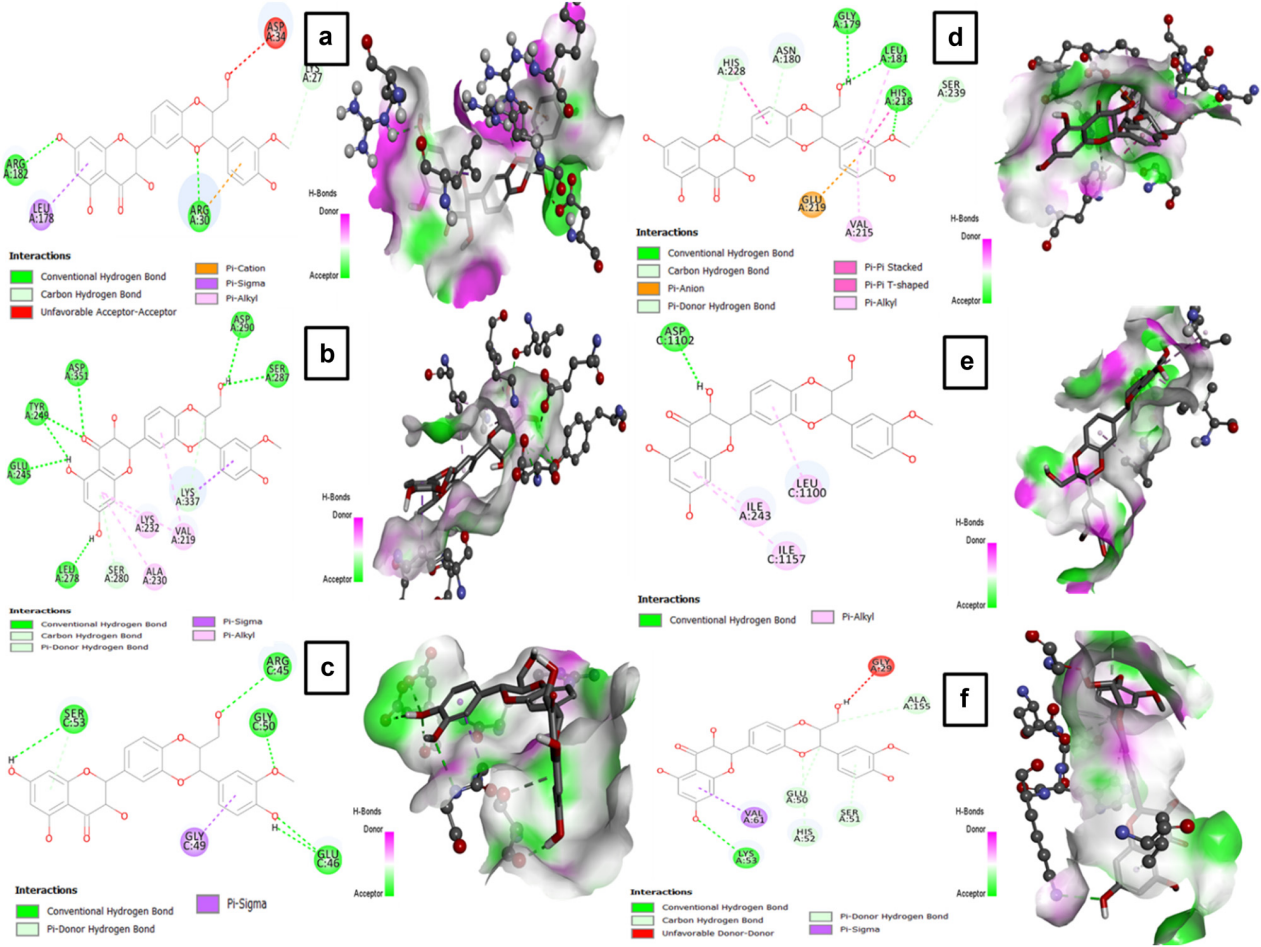
2D and 3D interaction of SIL with target proteins (a) IL-6 (1ALU), (b) TGF-β1 (3KFD), (c) COL1A1 (5cvb), (d) MMP-1 (1hfc), (e) TIMP-1 (2e2d), and (f) GPX-7 (2gh5).

In the docking results, the binding conformation with the lowest free energy indicates the highest binding affinity. The molecular docking studies revealed strong interaction of CT with target proteins IL-6, TGF-β, TIMP-1, MMP-1, and GPx-1 through hydrogen bonds having binding affinities −4.5, −6.4, −6.1, −5.7 and −5.6, respectively (Table 2). On the contrary, no hydrogen bond was formed between COL1A1 and CT. Interactions between Standard drug SIL and target proteins IL-6, TGF-β, TIMP-1, COL1A1, MMP-1, and GPx-1 are shown in Table 2. The binding affinities between these target proteins and SIL were −7.0, −10.6, −8.8, −7.3, −9.8, and −9.2 respectively. 2D and 3D interaction of CT and SIL with target proteins has been presented in Figures 8 and 9.

**Table 2:** Binding energies and interacting residues of ligands and protein after molecular docking

Ligands	Target protein	Binding affinity	Hydrogen bond
CT	IL-6 (1ALU)	−4.5	Leu 64
	TGF-β1 (3KFD)	−6.4	Asp 333
	TIMP-1 (2e2d)	−6.1	Pro 242
	COL1A1 (5cvb)	−4.1	0
	MMP-1 (1hfc)	−5.7	Arg 214, Tyr 237
	GPX-7 (2gh5)	−5.6	Ser 470
SIL	IL-6 (1ALU)	−7	Arg 30, Arg 182
	TGF-β1 (3KFD)	−10.6	Asp 351, Tyr 249, Glu 245, Leu 278, Asp 290, Ser 287
	TIMP-1 (2e2d)	−8.8	Asp 1102
	COL1A1 (5cvb)	−7.3	Arg 45, Glu 46, Gly 50, Ser 53
	MMP-1 (1hfc)	−8.8	Leu 181, Gly 179, His 218
	GPX-7 (2gh5)	−9.2	Lys 53

## 3 Discussion

The liver is the major organ implicated in maintaining energy homeostasis mainly via lipid and glucose metabolism. Besides, numerous xenobiotics and drugs are detoxified by the liver. The predominant role played by the liver in the transformation and elimination of chemical agents from the body renders it vulnerable to drug-induced damage. Any injury to liver can lead to serious health complications [25]. There is a dire need for hepatoprotective agents that are safer and more effective. The hepatoprotective potential of medicinal plants is highlighted through various studies [26]. CT is a monoterpenes alcohol and is present in the essential oil of plants belonging to the genus Cymbopogon. Several pharmacological properties of CT have been reported including anti hyperalgesia, anti-convulsant, and orofacial anti-nociception [27]. Considering the need to find new drug sources with enhanced efficacy and reduced side effects, and acknowledging the advantageous pharmacological effects of CT, this research aimed to determine its hepatoprotective potential. In this study, we used human hepatoma cell lines Hep2G to probe the hepatoprotective potential of CT. HepG2 has been widely used in toxicity screening and mechanistic studies, owing to its high stability, unlimited life span, and ready availability [28]. In the present work, we used Hep2G cell lines for experimental evaluation of cell viability in liver cells under the influence of ethanol and treated drugs.

HepG2 cells when treated with ethanol (8%) for 24 h exhibited a 50% decrease in cell viability that has been corroborated in numerous studies [29]. On the contrary, CT enhanced cells’ viability indicated by several assays performed. DAPI and PI fluorescent staining also displayed an increased number of viable cells in the CT-treated group as compared to the ethanol-treated group, thus demonstrating its cytoprotective potential.

The molecular docking technique helps in simulating the interaction between a small molecule and a protein on a detailed atomic level. This enables the characterization of how small molecules behave in the binding site of target proteins and helps in understanding essential biochemical processes [30]. SIL is known for its liver-protecting properties and is used to treat various liver conditions. Research shows that it has strong antioxidant properties that help shield the liver from damage [31]. We used SIL as a reference drug to compare interactions between the target molecule and CT with interactions between SIL and the target. During *in silico* studies, we observed strong interaction of CT with target proteins IL-6, TGF-β, TIMP-1, MMP-1, and GPx-1 through hydrogen bonds that were comparable to SIL.

To further validate the results of molecular docking the relative expression of these inflammatory biomarkers, IL-6, TGF-β1, COL1A1, MIMP-1, TIMP-1, and GPX-7 were assessed. IL-6 is a type of pleiotropic cytokine that impacts a wide range of cells. Human TH17 differentiation and IL-17 production are regulated by IL-6, which is crucial to the liver inflammation caused by ethanol. Both patients with acute and chronic liver disorders have markedly raised levels of IL-6. Moreover, patients with cirrhosis or alcoholic hepatitis have been shown to have elevated blood concentrations of IL-6 [32,33]. Our study also found that the diseased group had higher expression of IL-6 while treatment with CT dramatically reduced IL-6 expression levels, demonstrating its protective function.

Alcohol-induced inflammation upregulates TGF-β1 expression and is thought to be a central regulator of pathology and progression of alcoholic liver disorders [34,35]. In liver fibrosis, TGF-β1 expression is increased, and liver cells including Kupffer cells and HSCs release TGF-β1 [36]. By the suppression of DNA methyltransferase (DNMT) 1 and DNMT3a expressions as well as overall DNMT activity, TGF-βI can cause overexpression of COL1A1. One study indicates that a key mechanism in controlling the TGF-β1-induced COL1A1 gene expression is the DNMT-mediated DNA methylation [37]. As a result, elevated TGF-β1 can cause liver fibrosis by increasing the expression rate of COL1A1 [38,39,40]. Our findings also showed that the disease sample had higher levels of TGF-1 and COL1A1, whereas CT decreased their expression, demonstrating its anti-fibrotic potential.

MMP-1 is a protein that breaks down collagen, gelatin, laminin, and complement C1q, as well as other ECM and non-ECM substrates. As a result, MMP-1 contributes to the fibrotic and inflammatory processes [41]. According to studies, MMP-1 overexpression reduces fibrosis by encouraging collagenase-1 degradation, changes the ECM network and subsequently the interaction between cells and the ECM, stimulates hepatocyte proliferation and consequently liver regeneration, and encourages HSC autophagy and consequently lowers synthesis of collagen [42]. Moreover, MMP-1 is essential for the breakdown of ECM while experimental liver fibrosis is recovering [43]. Our research showed that MMP-1 levels were upregulated in the disease group, which suggested that elevated MMP-1 levels might be counteracting ethanol-related damage. MMP-1 levels were decreased in the CT-treated groups, which may have been caused by lessened cellular damage. The level of TIMP-1 is noticeably higher in liver fibrosis. It is thought that TIMP-1 encourages fibrosis in the wounded liver by inhibiting MMP and degrading the extracellular matrix in hepatic fibrogenesis [44]. TIMP-1 is primarily produced by HSC, and persistent TIMP-1 expression is now considered an essential factor contributing to the development of fibrosis [45,46,47]. Our findings showed elevated TIMP-1 expression in the diseased group, which is consistent with earlier research, and CT-protected cells by suppressing TIMP-1.

Besides implication of oxidative stress in triggering liver fibrosis is obvious from previous studies conducted [48,49]. A well-known controller of ROS-dependent oxidative DNA damage is GPX7 [50]. The fact that GPX7 is overexpressed in response to HSC activation and that it controls the production of pro-fibrotic genes by controlling intracellular ROS levels suggests that oxidative stress participates in the development of liver fibrosis through a progression of NASH [51]. Our research also showed that GPX7 expression increased in ethanol-impaired HepG2 cells and CT-treated groups demonstrated a downregulation in GPX7, indicating the protective effect of CT against oxidative stress.

## 4 Conclusion

It can be concluded that CT possesses immunomodulatory, anti-inflammatory, and anti-oxidant potential, which may be attributed to the downregulation of inflammatory and ECM-modulating biomarkers. Strong interaction between CT and IL-6, TGF-β, TIMP-1, MMP-1, and GPx-1 through hydrogen bonding in *in silico* studies shows its propensity to modulate these target proteins. Moreover, CT protected HepG2 cells against ethanol-mediated cell death, owing to its anti-oxidant, immunomodulatory, and hepatoprotective effects. However, further research is warranted to evaluate the precise mechanism of action and pharmacokinetic and pharmacodynamic profiles of CT.
